# Association between leisure motivation and continuous participation intention among Chinese ultimate frisbee participants: exploring the roles of leisure involvement and information acquisition

**DOI:** 10.3389/fspor.2026.1797171

**Published:** 2026-06-03

**Authors:** Shengguo Tian, Dawei Zhang, Chunhua Zhang, Lichuan Zhang, Yunjing Guo, Mingkun Ma

**Affiliations:** 1 School of Sports and Health Engineering, Hebei University of Engineering, Handan, Hebei, China; 2 School of P. E., Shanxi Normal University, Taiyuan, Shanxi, China; 3 Department of Sports Training, Shanxi Sports Vocational College, Taiyuan, Shanxi, China; 4 School of P. E., Nanjing Xiaozhuang University, Nanjing, Jiangsu, China; 5 Institute of Physical Education and Health, Yulin Normal University, Yulin, Guangxi, China

**Keywords:** continuous participation intention, information acquisition, leisure involvement, leisure motivation, ultimate frisbee

## Abstract

**Introduction:**

Understanding continuous participation intention in emerging leisure sports such as Ultimate Frisbee is important for promoting leisure-time physical activity. Drawing on leisure motivation theory and Social Information Processing theory, this study examines the relationships among leisure motivation, leisure involvement, information acquisition, and continuous participation intention among Chinese Ultimate Frisbee participants. The research aims to provide a clearer explanatory perspective for understanding sustained participation in emerging leisure sports and to offer practical implications for promoting participation in community-oriented leisure sports.

**Methods:**

Using snowball sampling, an online questionnaire survey was conducted with 724 Ultimate Frisbee participants from multiple regions in China. Data were analyzed using SPSS 27.0. Regression analyses were first conducted to examine the relationships among the study variables. PROCESS Model 6 with 5,000 bootstrap resamples was then used to test the indirect associations.

**Results:**

The results showed that leisure motivation was positively associated with continuous participation intention. Leisure involvement and information acquisition each showed significant indirect associations in the relationship between leisure motivation and continuous participation intention. In addition, a significant serial indirect association through leisure involvement and information acquisition was observed. Overall, a substantial proportion of the relationship between leisure motivation and continuous participation intention was reflected in these indirect associations.

**Discussion:**

The findings provide empirical evidence for understanding continuous participation intention in Ultimate Frisbee and also highlight the importance of leisure involvement and information acquisition in community-oriented leisure sports. In the context of the internet era, further improving the accessibility and effectiveness of Ultimate Frisbee-related information will help enhance individuals' continuous participation intention.

## 1 Introduction

How individuals form and maintain health-promoting behaviors during leisure time has been a central concern in contemporary psychology and behavioral science. A growing body of research indicates that leisure activities not only provide important contexts for acquiring skills, knowledge, and experience, but also serve as platforms for self-expression and social interaction. Leisure participation is beneficial to individuals' positive affect, subjective well-being, and broader psychosocial functioning (1–3). At the same time, regular physical activity is important for promoting individuals’ physical and mental health, and is further related to quality of life and overall well-being (4–6). Accordingly, leisure sports—which combine physical engagement with psychological regulation—are increasingly recognized as an important life context associated with coordinated physical and mental health during leisure time (7–10).

Among the diverse forms of leisure sports, emerging activities such as disc sports (e.g., Ultimate Frisbee) have gained increasing public visibility in recent years. Compared with traditional competitive sports, Ultimate Frisbee has rapidly grown in popularity among young adults due to its moderate physical intensity, low barriers to entry, and strong emphasis on social interaction (11). According to publicly available media reports, the number of Ultimate Frisbee participants in China has increased markedly in recent years. Alongside this growth in participation, scholarly interest has expanded across multiple domains. Existing research has examined issues such as injury prevention and risk management (12, 13), competition rules and ethical norms (14, 15), facility use and spatial governance (16), development and promotion pathways of disc sports (17), and social–psychological influences embedded in participation contexts, including teamwork, communication, and cultural norms (11). Collectively, these studies provide an important theoretical foundation for the standardization and sustainable development of Ultimate Frisbee. Nevertheless, from the perspective of long-term and stable engagement, existing research has tended to focus on participation outcomes or isolated correlates—such as participation frequency, behavioral intention, or health-related benefits—while paying relatively limited attention to the psychological and contextual factors associated with sustained participation.

In contemporary society, the widespread integration of the internet and social media into everyday life has reshaped how individuals acquire information, engage in social interaction, and make leisure-related decisions (18). In community-oriented leisure sports such as Ultimate Frisbee, participation increasingly takes place within social environments shaped by club communication, peer networks, social media interaction, and shared cultural norms (19, 20). In this context, sustained participation intention may be associated not only with internal motivational factors but also with how participants perceive and engage with activity-related information in these social environments (21). Social Information Processing (SIP) theory holds that individuals do not form judgments in isolation. Rather, they selectively attend to, interpret, and integrate informational cues embedded in their social contexts, thereby constructing cognitive evaluations of behavioral outcomes, which in turn inform the formation of attitudes and intentions (22). Prior research further indicates that information environments encountered during leisure and social activities are associated with participation-related judgments and intentions (23–26). Despite these insights, empirical research has devoted relatively limited attention to the role of information acquisition within interest-driven, voluntary, and highly interactive leisure sport contexts such as Ultimate Frisbee. Therefore, in the context of Ultimate Frisbee, the relationship between information acquisition and individuals' continuous participation intention still warrants further examination.

Building on this rationale, the present study focuses on Chinese Ultimate Frisbee participants and examines continuous participation intention from an integrative perspective incorporating both psychological and information-related factors. Specifically, this study examines whether leisure motivation, leisure involvement, and information acquisition are associated with individuals' intentions to maintain long-term participation in this emerging leisure sport. By empirically examining these relationships, the study aims to provide further evidence for understanding sustained participation in Ultimate Frisbee and similar community-based leisure sports, while also offering practical implications for participation support in such contexts.

## 2 Theoretical background and hypotheses

### 2.1 Leisure motivation and continuous participation intention

Leisure motivation is commonly regarded as an important psychological factor related to participation in leisure activities and is often used to understand why individuals choose to take part in a particular activity (27, 28). In leisure and sport contexts, individuals with higher levels of leisure motivation are more likely to perceive the activity as personally meaningful and worthwhile, which is associated with a stronger intention to continue participation. Prior research has shown that leisure motivation is positively related to continued engagement and participation intention in leisure and sport settings (29–31). Accordingly, in the context of Ultimate Frisbee, stronger leisure motivation may be associated with higher levels of continuous participation intention. Based on this, the following hypothesis is proposed:

H1: Leisure motivation is positively associated with continuous participation intention.

### 2.2 The mediating role of leisure involvement

Leisure involvement is commonly conceptualized as a relatively enduring state of psychological engagement that develops through sustained participation in a specific leisure or sport activity (32–34). Compared with leisure motivation, which may vary across situations or participation phases, leisure involvement places greater emphasis on a more stable sense of identification with the activity and sustained psychological attachment (28).

Previous research has shown that individuals with higher levels of leisure motivation tend to report more positive leisure involvement (35, 36). Higher levels of leisure involvement are, in turn, associated with stronger re-participation intention (37, 38). Taken together, these findings suggest that as participants' leisure motivation increases, their leisure involvement may also become stronger, which may further be associated with higher levels of continuous participation intention (39). Accordingly, in the context of Ultimate Frisbee, participants with stronger leisure motivation may report higher levels of leisure involvement, and greater leisure involvement may be associated with stronger continuous participation intention. In this sense, leisure involvement may serve as an important psychological link between leisure motivation and continuous participation intention. Based on this, the following hypothesis is proposed:

H2: Leisure involvement mediates the relationship between leisure motivation and continuous participation intention.

### 2.3 The mediating role of information acquisition

Information acquisition refers to individuals' subjective perceptions of the accessibility and quality of activity-related information encountered during Ultimate Frisbee participation. Prior research on information behavior suggests that motivation is closely related to information acquisition and information seeking (40–42). Individuals do not form behavioral judgments in isolation. Rather, they construct perceptions, attitudes, and behavioral intentions by drawing on informational cues embedded in their social environments (22, 43). In the context of Ultimate Frisbee, participants with stronger leisure motivation may pay greater attention to activity-related information available in the participation environment and may develop more positive perceptions of that information. When such information is perceived as more accessible and of higher quality, continuous participation intention may also be stronger (44, 45). In this sense, information acquisition may be an important informational factor linking leisure motivation and continuous participation intention. Therefore, the present study proposes that information acquisition may account for part of the association between leisure motivation and continuous participation intention. Based on this, the following hypothesis is proposed:

H3: Information acquisition mediates the relationship between leisure motivation and continuous participation intention.

### 2.4 The serial indirect association of leisure involvement and information acquisition

Building on the preceding hypotheses, the present study further considers whether leisure motivation, leisure involvement, information acquisition, and continuous participation intention may be linked in a serial pattern. Existing theory and prior findings suggest a relatively plausible ordering, namely that leisure motivation is associated with leisure involvement. Individuals with higher levels of involvement usually pay greater attention to relevant cues in the participation context and more actively encounter and perceive activity-related information (46, 47). From a social information processing perspective, when such information is perceived as more accessible and of higher quality, continuous participation intention becomes stronger (22, 48). However, direct evidence placing leisure motivation, leisure involvement, information acquisition, and continuous participation intention within the same analytical framework remains relatively limited. In addition, based on cross-sectional data, other possible orderings or reciprocal associations cannot be adequately distinguished. Taken together, the existing literature provides a theory-informed rationale for specifying leisure involvement before information acquisition in the serial model. Based on this, the following hypothesis is proposed:

H4: Leisure involvement and information acquisition mediate the relationship between leisure motivation and continuous participation intention.

**Figure 1:**
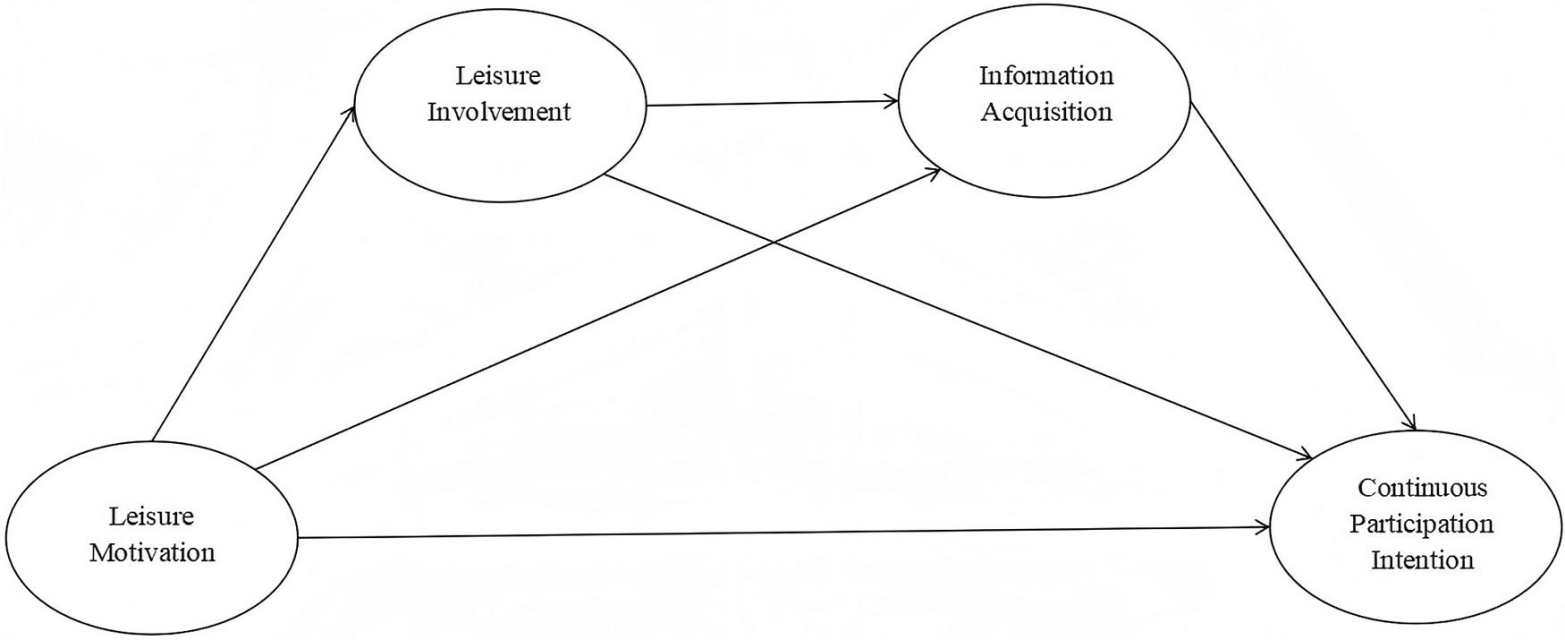
Hypothesized a mediation model.

Taken together, the above hypotheses form the conceptual framework of the present study, which depicts the associations among leisure motivation, leisure involvement, information acquisition, and continuous participation intention (Figure 1).

## 3 Materials and methods

### 3.1 Participants

The participants in the present study were Ultimate Frisbee participants from different regions of China, with the sample mainly drawn from Beijing, Shanghai, Shanxi Province, and Shaanxi Province. These regions were selected for two main reasons. First, they are located in eastern, central, and western China, respectively, thus providing a certain degree of geographic diversity and helping to enhance the diversity of the sample source. Second, Ultimate Frisbee has developed relatively early in these regions, where club organizations, competitive events, and participation networks are comparatively mature, providing a more stable practical basis for questionnaire distribution and data collection.

After approval was obtained from the Ethics Committee of Shanxi Normal University, data were collected using a snowball sampling approach, as Ultimate Frisbee participants do not constitute a fixed population and random sampling was difficult to implement. The survey was administered in the form of an online questionnaire. Data collection proceeded in two steps. First, the researchers identified initial participants with Ultimate Frisbee participation experience by visiting student clubs on campus and community-based club organizations. Second, these initial participants were asked to further distribute the questionnaire within their own communities or organizations in order to expand sample coverage. All participants voluntarily took part in the survey after providing informed consent. A total of 792 questionnaires were distributed, of which 724 were considered valid, yielding an effective response rate of 91.41%. Of the participants, 396 were female (54.70%) and 328 were male (45.30%). Regarding age, 202 participants (27.90%) were under 20 years old, 254 (35.08%) were aged 20–25 years, 126 (17.40%) were aged 26–30 years, 87 (12.02%) were aged 31–35 years, and 55 (7.6%) were aged 36 years or older. With respect to participation experience, 271 participants (37.43%) reported less than one year of Ultimate Frisbee participation, 221 (30.53%) reported one to two years, and 232 (32.04%) reported two years or more.

### 3.2 Measurement

#### 3.2.1 Leisure motivation

The present study measured leisure motivation using the Leisure Motivation Scale originally developed by Beard and Ragheb (27) and subsequently adapted and validated in Chinese contexts by Jin (7) and Li et al. (49). The scale comprises four dimensions—intellectual motivation, social motivation, competence–mastery motivation, and stimulus–avoidance motivation—and includes 16 items in total, such as“to learn and experience Ultimate Frisbee”and“to improve my social skills.”All items were rated on a 5-point Likert scale ranging from 1 (strongly disagree) to 5 (strongly agree). Because the present study focused on the overall level of leisure motivation, and after confirming the reasonableness of the scale's dimensional structure, item scores were summed to generate a total leisure motivation score, with higher scores indicating stronger leisure motivation. In this study, the scale demonstrated good internal consistency (Cronbach's *α* = 0.805). To further assess structural adequacy, the Kaiser–Meyer–Olkin (KMO) test and Bartlett's test of sphericity were conducted. The results showed a KMO value of 0.846, and Bartlett's test was significant (*χ*² = 5,850.508, df = 120, *p* < 0.001), confirming that the scale demonstrated good validity.

#### 3.2.2 Leisure involvement

The present study measured leisure involvement using the scale proposed by Kyle et al. (33) and subsequently adapted and validated in Chinese contexts by Geng et al. (50) and Shao et al. (51). The scale consists of five items, such as “Ultimate Frisbee is the sport I most want to do in my leisure time” and “I always look forward to participating in Ultimate Frisbee.” All items were rated on a 5-point Likert scale ranging from 1 (strongly disagree) to 5 (strongly agree). Item scores were summed to generate a total leisure involvement score, with higher scores indicating a higher level of leisure involvement. In this study, the scale demonstrated good internal consistency (Cronbach's *α* = 0.884). To further assess structural validity, the KMO test and Bartlett's test of sphericity were conducted. The results showed a KMO value of 0.886, and Bartlett's test was significant (*χ*² = 1,808.036, df = 10, *p* < 0.001), suggesting that the scale demonstrated acceptable structural adequacy.

#### 3.2.3 Information acquisition

The information acquisition scale used in this study was developed with reference to measures applied in related studies by Zhang (52) and Zheng (24) in Chinese contexts. The scale consists of six items, such as “The Ultimate Frisbee-related information I obtain can meet my actual needs” and “I can obtain Ultimate Frisbee-related information relatively easily.” All items were rated on a 5-point Likert scale ranging from 1 (strongly disagree) to 5 (strongly agree). Item scores were summed to generate a total information acquisition score, with higher scores indicating a higher level of information acquisition. In this study, the scale demonstrated good internal consistency (Cronbach's *α* = 0.779). To further assess structural validity, the KMO test and Bartlett's test of sphericity were conducted. The results showed a KMO value of 0.756, and Bartlett's test was significant (*χ*² = 1,494.513, df = 15, *p* < 0.001), suggesting that the scale demonstrated acceptable structural adequacy.

#### 3.2.4 Continuous participation intention

The present study measured continuous participation intention using the scale proposed by Bitner (53) and subsequently adapted and validated in Chinese contexts by Li et al. (49) in the context of sport participation. The scale consists of three items, such as “I intend to continue participating in Ultimate Frisbee.” All items were rated on a 5-point Likert scale ranging from 1 (strongly disagree) to 5 (strongly agree). Item scores were summed to generate a total continuous participation intention score, with higher scores indicating stronger continuous participation intention. In this study, the scale demonstrated good internal consistency (Cronbach's *α* = 0.787). To further assess structural validity, the KMO test and Bartlett's test of sphericity were conducted. The results showed a KMO value of 0.692, and Bartlett's test was significant (*χ*² = 646.714, df = 3, *p* < 0.001), suggesting that the scale demonstrated acceptable structural adequacy.

#### 3.2.5 Construct validity

An exploratory factor analysis (principal axis factoring with varimax rotation) was conducted to assess construct validity. The KMO value was 0.888 and Bartlett's test was significant (*χ*² = 10,796.70, df = 435, *p* < 0.001). The extracted factors were consistent with the theorized constructs; all items loaded primarily on their intended factor (loadings = 0.739–0.860), and no substantial cross-loadings were observed.

### 3.3 Statistical analysis

Participants completed the questionnaire independently under a unified set of instructions and in a distraction-free environment to enhance response accuracy. All responses were anonymous and used solely for academic research purposes. Following data collection, the data were coded and analyzed using SPSS 27.0. The analytical procedure consisted of several steps. First, common method bias was examined across all measurement items. Second, descriptive statistics were calculated for all study variables. Third, Pearson correlation analyses and multiple linear regression analyses were conducted to examine the relationships among the study variables. Finally, mediation and serial mediation effects were tested using the PROCESS macro (Model 6). Bootstrap resampling with 5,000 samples was used to generate bias-corrected confidence intervals for the indirect effects, thereby improving the robustness of the mediation analyses.

## 4 Results

### 4.1 Common method bias test

Given that all variables were measured via self-report, Harman's single-factor test was conducted to assess potential common method bias. The first unrotated factor accounted for 24.12% of the total variance, which was below the commonly used 40% threshold. These results suggest that common method bias was unlikely to pose a serious concern and that the data were appropriate for subsequent analyses.

### 4.2 Correlations among the variables

Pearson correlation analysis was conducted to examine the relationships among the study variables, and the results are presented in Table 1. The results showed that leisure motivation, leisure involvement, information acquisition, and continuous participation intention were all significantly and positively correlated, with correlation coefficients below 0.60. Gender, age, and years of participation were not significantly correlated with the core study variables. These findings suggest that the core variables were statistically associated, while the magnitude of the bivariate correlations did not indicate excessively high overlap among the predictors. To further assess collinearity, additional diagnostics were conducted. The variance inflation factor (VIF) values for all predictors were below 10, indicating that no serious multicollinearity was present. Therefore, the data were considered suitable for subsequent regression analyses.

**Table 1:** Correlations among the variables (*N* = 724).

Variables	1	2	3	4	5	6	7
Gender	1.000						
Age	0.223**	1.000					
Years of participation	0.085*	0.402**	1.000				
Leisure Motivation	−0.013	−0.054	−0.019	1.000			
Leisure Involvement	−0.002	−0.021	0.002	0.406**	1.000		
Information Acquisition	−0.013	0.027	−0.010	0.301**	0.496**	1.000	
Continuous Participation Intention	−0.055	−0.008	−0.028	0.279**	0.434**	0.537**	1.000

* *p* < 0.05.

** *p* < 0.01.

### 4.3 Regressions among the variables

Regression analyses were conducted to examine the relationships among leisure motivation, leisure involvement, information acquisition, and continuous participation intention. As shown in Table 2, leisure motivation was positively associated with leisure involvement (*β* = 0.406, *t* = 11.939, *p* < 0.001). When information acquisition was specified as the dependent variable, both leisure motivation (*β* = 0.119, *t* = 3.399, *p* < 0.01) and leisure involvement (*β* = 0.447, *t* = 12.741, *p* < 0.001) were positively associated with information acquisition. With regard to continuous participation intention, leisure motivation (*β* = 0.073, *t* = 2.177, *p* < 0.05), leisure involvement (*β* = 0.197, *t* = 5.342, *p* < 0.001), and information acquisition (*β* = 0.417, *t* = 11.763, *p* < 0.001) were all positively associated with continuous participation intention. Taken together, these findings are consistent with the hypothesized positive associations among leisure motivation, leisure involvement, information acquisition, and continuous participation intention, and provide a basis for the subsequent mediation and serial mediation analyses.

**Table 2:** Regression analyses for the study variables.

Variables	LI			IA			CPI		
	*β*	*t*	95% CI	*β*	*t*	95% CI	*β*	*t*	95% CI
LM	0.406	11.939***	0.527, 0.734	0.119	3.399**	0.072, 0.270	0.073	2.177*	0.009, 0.176
LI				0.447	12.741***	0.349, 0.476	0.197	5.342***	0.101, 0.219
IA							0.417	11.763***	0.305, 0.427
R	0.406			0.508			0.574		
R^2^	0.165			0.258			0.330		
Adjusted R^2^	0.164			0.256			0.327		
F	142.548***			125.161***			118.002***		

LM, leisure motivation; LI, leisure involvement; IA, information acquisition; CPI, continuous participation intention.

* *p* < 0.05.

** *p* < 0.01.

*** *p* < 0.001.

### 4.4 Direct and indirect associations

To further examine the associations between leisure motivation and continuous participation intention among Ultimate Frisbee participants, mediation analyses were conducted using the PROCESS macro (Model 6) with 5,000 bootstrap resamples. Leisure motivation was specified as the independent variable, continuous participation intention as the dependent variable, and leisure involvement and information acquisition as the mediators. The results are presented in Table 3. The total association between leisure motivation and continuous participation intention was 0.351 (SE = 0.045, 95% CI [0.263, 0.440]). The direct association between leisure motivation and continuous participation intention was 0.092 (SE = 0.042, 95% CI [0.009, 0.175]), accounting for 26.21% of the total association.

**Table 3:** Bootstrapped indirect and serial indirect associations.

Effect type	Effect (B)	Bootstrap SE	95% CI (LLCI)	95% CI (ULCI)	Proportion (%)
Total effect (LM→ CPI)	0.351	0.045	0.263	0.440	—
Direct effect (LM → CPI)	0.092	0.042	0.009	0.175	26.21%
Total indirect effect	0.259	0.031	0.201	0.322	73.79%
LM→LI→CPI	0.101	0.024	0.056	0.151	28.77%
LM→IA→CPI	0.063	0.020	0.026	0.102	17.95%
LM→LI→IA→CPI	0.095	0.013	0.071	0.123	27.07%

LM, leisure motivation; LI, leisure involvement; IA, information acquisition; CPI, continuous participation intention.

The total indirect association between leisure motivation and continuous participation intention was 0.259 (SE = 0.031, 95% CI [0.201, 0.322]), accounting for 73.79% of the total association. The indirect association through leisure involvement was 0.101 (SE = 0.024, 95% CI [0.056, 0.151]), accounting for 28.77% of the total association. The indirect association through information acquisition was 0.063 (SE = 0.020, 95% CI [0.026, 0.102]), accounting for 17.95% of the total association.

The serial indirect association through leisure involvement and information acquisition was 0.095 (SE = 0.013, 95% CI [0.071, 0.123]), accounting for 27.07% of the total association. Because the 95% bootstrap confidence intervals for the total indirect association and all specific indirect associations did not include zero, these indirect associations were considered statistically significant. Overall, the findings suggest that the relationship between leisure motivation and continuous participation intention was reflected in both direct and indirect associations.

## 5 Discussion

The present study examined the relationship between leisure motivation and continuous participation intention in a leisure sport context. It further analyzed the indirect associations reflected through leisure involvement and information acquisition. The findings showed that leisure motivation, leisure involvement, information acquisition, and continuous participation intention were all positively related. In addition, the observed indirect associations suggest that leisure involvement and information acquisition may jointly help explain the relationship between leisure motivation and continuous participation intention in the context of Ultimate Frisbee.

### 5.1 Direct association between leisure motivation and continuous participation intention

Hypothesis H1 was supported, indicating that leisure motivation was positively associated with continuous participation intention. This finding is broadly consistent with previous research. Alexandris et al. (54) reported a significant association between leisure motivation and intention to continue participation in a recreational skiing context. Choi (30) further found a positive relationship between participation motivation and continuous participation intention among university futsal club participants. Ji and Wu (31) also showed that exercise motivation was significantly associated with continuous participation intention among Chinese CrossFit participants. Notably, the direct association between leisure motivation and continuous participation intention was relatively modest in the present study, suggesting that leisure motivation alone may not fully account for individuals' continuous participation intention. This pattern may be related to the characteristics of Ultimate Frisbee itself. As a leisure sport characterized by social interaction, participatory experience, and teamwork, Ultimate Frisbee enthusiasts'continuous participation intention may not depend solely on their initial leisure motivation, but may also be jointly associated with other proximal psychological variables that emerge during participation (55–57).

### 5.2 Leisure involvement in the relationship between leisure motivation and continuous participation intention

The present study found that leisure involvement showed a significant indirect association in the relationship between leisure motivation and continuous participation intention, thus supporting Hypothesis H2. Leisure involvement theory conceptualizes leisure involvement as an enduring form of psychological investment in an activity (32). From a theoretical perspective, prior research has provided support for different segments of this associative chain. Alexandris (35) found a positive association between motivation and the development of sport involvement in a recreational tennis context. Chen et al. (36) likewise reported a significant positive association between leisure motivation and leisure involvement among adolescents. In addition, Morris et al. (58) further showed that motivation and involvement were stably related among participants in high-risk leisure activities. At the same time, prior research has also indicated that higher levels of leisure involvement are generally associated with stronger loyalty and continued participation tendencies (28). Wang et al. (37) found that leisure involvement was positively related to repurchase intention in the context of Chinese fitness clubs. Karakullukcu et al. (38) further reported that, among recreational tennis participants, higher involvement was associated with stronger re-participation intention. Although prior studies directly examining leisure motivation, leisure involvement, and continuous participation intention within the same model remain relatively limited, these findings provide support for understanding leisure involvement as an important intervening variable in the relationship between leisure motivation and continuous participation intention. In the context of Ultimate Frisbee, participants may enter the activity with initial motivation and gradually develop stronger psychological investment, activity identification, and perceived personal importance during participation, which may in turn be reflected in stronger continuous participation intention.

### 5.3 Information acquisition in the relationship between leisure motivation and continuous participation intention

The present study found that information acquisition showed a significant indirect association in the relationship between leisure motivation and continuous participation intention, thus supporting Hypothesis 3. Social Information Processing theory suggests that individuals do not form behavioral judgments in isolation. Rather, they construct perceptions, attitudes, and behavioral intentions by drawing on informational cues embedded in their social environments (22, 43). In this sense, the present findings provide further empirical evidence for understanding informational factors in leisure sport contexts.

It is important to note that information acquisition in the present study primarily refers to individuals' subjective perceptions of the accessibility and quality of activity-related information encountered during Ultimate Frisbee participation (59, 60). Shen et al. (41) argued that motivation can serve as an important perspective for understanding information-seeking behavior, because individuals' motivational states influence whether and to what extent they actively attend to and acquire information. In a leisure sport such as Ultimate Frisbee, which is characterized by strong social interaction and community embeddedness, information about training arrangements, competition opportunities, community activities, and participation norms is often encountered and perceived through peer interaction, social media, and club communication. Prior research has shown that information quality and related perceptions are positively associated with continuance intention or sustained participation tendency (24, 61–63). Therefore, in the context of Ultimate Frisbee, when participants perceive relevant information as more accessible and of higher quality, their continuous participation intention may also be stronger.

From the perspective of the present findings, information acquisition is not the only factor that helps explain the relationship between leisure motivation and continuous participation intention. However, its indirect association suggests that the informational context may be an important aspect that should not be overlooked in understanding this relationship. In other words, participants with stronger leisure motivation may form more positive perceptions of activity-related information during participation, and such perceptions may in turn be associated with stronger continuous participation intention.

### 5.4 The serial indirect association of leisure involvement and information acquisition

The present findings indicate that the relationship between leisure motivation and continuous participation intention was reflected not only in two relatively independent indirect associations, namely through leisure involvement and information acquisition, but also in the serial indirect association formed by these two variables. From a theoretical perspective, individuals with stronger leisure motivation may develop higher levels of leisure involvement during participation (64, 65). In the context of Ultimate Frisbee, when participants show stronger leisure motivation, they may gradually develop higher levels of psychological investment, activity identification, and perceived personal importance. The present study further suggests that the level of leisure involvement may also be related to individuals' information acquisition about the activity. On the one hand, individuals with higher leisure involvement tend to pay greater attention to relevant cues in the activity context. On the other hand, they are also more likely to actively encounter and perceive activity-related information (46, 47). This is closely related to the strong social interaction and community embeddedness of Ultimate Frisbee itself. At the same time, prior research has shown that individuals' perceptions of information accessibility and information quality are associated with their evaluations of activity value, participation experience, and subsequent behavioral judgments (66).

This suggests that when participants, on the basis of higher leisure involvement, form more positive perceptions of Ultimate Frisbee-related information, their continuous participation intention may also be stronger. As the internet and social media become increasingly integrated into daily life, individuals are more likely to continuously encounter and perceive activity-related information during participation in leisure sports. Therefore, information acquisition should be regarded as an important aspect that should not be overlooked in understanding continuous participation intention in Ultimate Frisbee.Taken together, these findings provide contextualized support for leisure involvement theory and Social Information Processing theory, and help explain patterns of sustained participation in interest-driven, voluntary, and community-oriented leisure sports such as Ultimate Frisbee.

## 6 Conclusion

This study focused on Chinese Ultimate Frisbee participants and examined the relationships among leisure motivation, leisure involvement, information acquisition, and continuous participation intention, as well as the indirect associations among these variables. Based on the findings, three conclusions can be drawn. First, leisure motivation was positively associated with continuous participation intention. After leisure involvement and information acquisition were included in the model, this direct association remained significant, indicating that the relationship was reflected in both direct and indirect associations. Second, both leisure involvement and information acquisition showed significant indirect associations. Third, leisure motivation was also associated with continuous participation intention through the serial indirect association formed by leisure involvement and information acquisition. Taken together, by incorporating both leisure involvement and information acquisition into a single analytical framework, this study extends previous leisure sport research and provides new empirical evidence for understanding continuous participation intention in Ultimate Frisbee. More broadly, these findings may also offer useful insights for interpreting sustained participation in other emerging, community-oriented, and interest-driven leisure sports beyond the specific context of Ultimate Frisbee.

## 7 Limitations and future directions

Although the present study provides empirical evidence for understanding continuous participation intention among Ultimate Frisbee participants, several limitations should be acknowledged. First, this study used a cross-sectional survey design. Although it was grounded in relevant theory and prior research, it still does not allow for precise causal inference. Future researchers are encouraged to adopt longitudinal designs and use cross-lagged panel analyses to further examine the relationship between leisure motivation and continuous participation intention. Second, given the characteristics of the target population, this study primarily used snowball sampling to distribute the questionnaire. Although this approach was practically feasible, it may not have fully captured the heterogeneity and diversity of Ultimate Frisbee participants, and the results may have been influenced by respondents' subjective states and the specific survey context.Moreover, because all variables were measured using same-source self-report data at a single time point, common method bias cannot be ruled out completely, and Harman's single-factor test provides only a limited assessment in this regard. Future studies could improve the representativeness and broader applicability of the findings by expanding regional coverage, including participants from more diverse participation backgrounds, and adopting more systematic sampling strategies where feasible. Third, the present study did not sufficiently consider potential constraints or barriers that may hinder participation in Ultimate Frisbee. Future research should explicitly examine inhibiting factors, including limited facility resources, low levels of public awareness, and insufficient access to professional guidance, in order to develop a more comprehensive understanding of the factors affecting the development and sustainability of Ultimate Frisbee and similar leisure sport activities. Finally, this study focused on continuous participation intention rather than actual continued participation behavior. Although continuous participation intention is an important proximal indicator for understanding behavioral maintenance, it is not equivalent to actual long-term participation behavior. Therefore, greater attention should be given to the possible intention-behavior gap. Future studies could incorporate more objective indicators, such as participation records, attendance frequency, or activity-tracking data, in order to provide a more comprehensive understanding of sustained participation in Ultimate Frisbee and similar leisure sport activities.

## Data Availability

The original contributions presented in the study are included in the article/Supplementary Material, further inquiries can be directed to the corresponding author/s.
